# Highly Efficient Extraction of ^212^ Pb/^212^Bi from the Decay Chain of ^232^Th‐Based on Anion Exchange in Bromide Medium

**DOI:** 10.1002/advs.74721

**Published:** 2026-03-09

**Authors:** Lifeng Chen, Wannian Feng, Yuezhou Wei, Xuexiang He, Shaoying Wang, Zhongyuan Zhou, Qiang Wu, Ningchao Zheng, Xiangbiao Yin, Wenlong Li, Lingling Su, Shunyan Ning

**Affiliations:** ^1^ School of Nuclear Science and Technology University of South China Hengyang P. R. China; ^2^ Key Laboratory of Advanced Nuclear Energy Design and Safety Ministry of Education Hengyang P. R. China; ^3^ Institute of Nuclear Fuel Cycle and Materials, School of Mechanical Engineering Shanghai Jiao Tong University Shanghai P. R. China; ^4^ Laboratory of Nuclear Energy Chemistry, Institute of High Energy Physics Chinese Academy of Sciences Beijing P. R. China

**Keywords:** ^212 ^Pb, ^212^Bi, ^232^Th, anion exchange, decay chain, radioisotope, separation

## Abstract

Although ^212^Pb and ^212^Bi have significant applications in targeted α‐nuclide therapy for malignant tumors, their use is limited by the scarcity of ^212^Pb/^212^Bi resources. To address this bottleneck problem, an advanced strategy for selectively extracting ^212^Pb and ^212^Bi from the decay chain of ^232^Th is first proposed by using an efficient porous silica‐supported anion exchanger in bromide medium. The selective adsorption mechanisms of the resin for Pb(II) and Bi(III) in bromide medium are first clarified through spectroscopic analyses and theoretical calculations. Using the novel method and exchanger, 2.07 MBq of ^212^ Pb is successfully separated from ^232^Th and its daughters, with a record‐breaking chemical yield of 88.2% and an estimated radionuclide purity of over 99.9%. Mechanism analysis suggests that Pb(II) and Bi(III) can form anionic complexes in the HBr medium, with [PbBr^3^]^−^ and [BiBr_5_]^2−^ being the main species captured; while Th(IV), Ac(III) and Ra(II) mainly exist as cationic forms and thus can't be fixed. To date, this represents the first report of successfully extracting ^212^Pb/^212^Bi from ^232^Th and its daughters using anion exchanger in bromide medium, as well as the mechanism illustrations, which enables the supply of ^212^Pb/^212^Bi directly from the abundant natural thorium resource to be possible.

## Graphical abstract

This work provides an effective strategy for selectively extracting ^212^Pb and ^212^Bi from the decay chain of ^232^Th as well as its mechanism illustration. Using the novel strategy, 2.07 MBq of ^212^Pb is successfully separated from ^232^Th and its daughters, which enables the supply of ^212^Pb/^212^Bi directly from the abundant natural thorium resource to be possible.

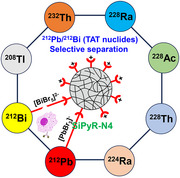

## 1 Introduction

Cancer has long been one of the most lethal diseases worldwide [1, 2, 3]. Due to frequent metastasis and recurrence, there remains a lack of specific drugs for cancer. Medical isotopes have unique advantages in cancer diagnosis and treatment. By facilitating the early detection and precise treatment of cancer, medical isotopes have significant implications for improving the cure rate of cancer. ^212^Pb/^212^Bi are important medical isotopes, which have attracted great attention worldwide in recent years [4, 5]. The half‐life of ^212^Pb is 10.6 h, and it can transform into ^212^Bi via β decay. ^212^Bi and its daughter products continuously release high‐energy, short‐range α particles during the decay process, which can efficiently kill cancer cells while minimizing damage to normal tissues and cells. They have shown excellent application potential and efficacy in targeted α‐nuclide therapy (TAT) for malignant tumors such as breast cancer, prostate cancer, and pancreatic cancer [6, 7, 8]. However, due to the scarcity of ^212^Pb/^212^Bi resources, the development of targeted drugs and clinical applications based on these isotopes are greatly limited [9, 10, 11]. Therefore, establishing novel and efficient methods and routes for obtaining ^212^Pb/^212^Bi resources is thus of great significance.

**FIGURE 1:**
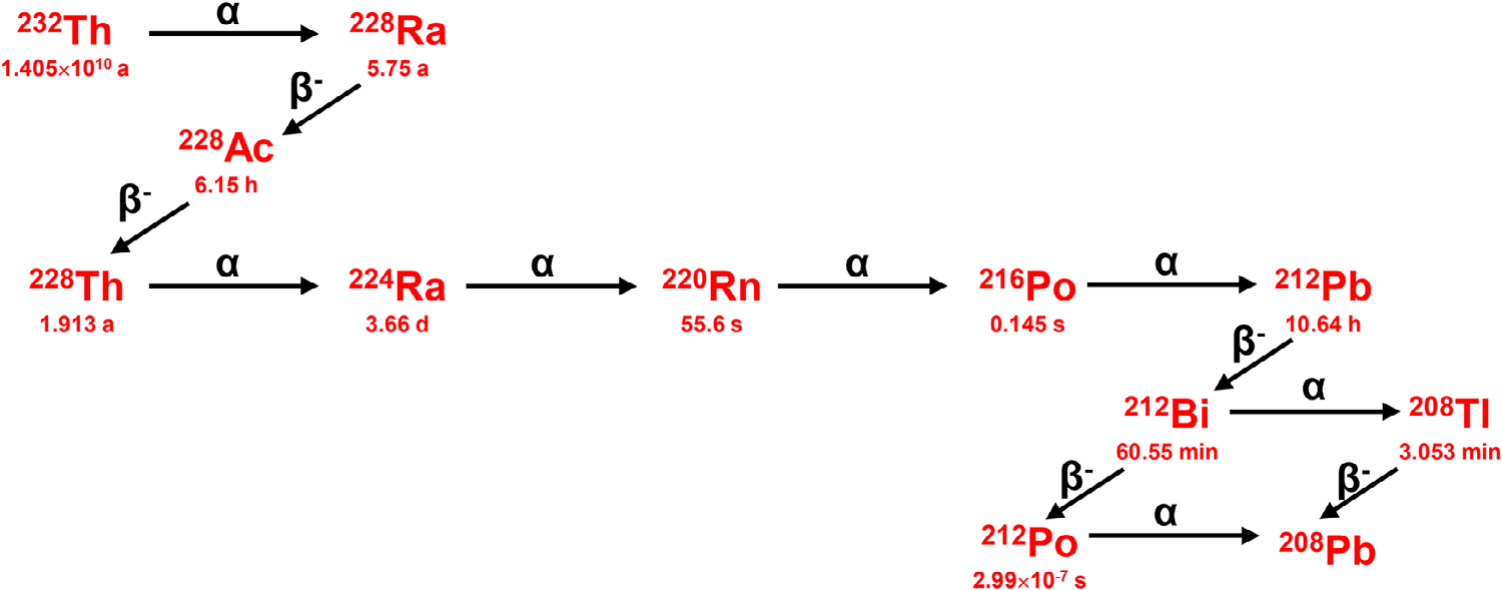
Decay chain of ^232^Th.

The preparation of ^212^Pb/^212^Bi currently mainly relies on two approaches: the ^228^Th/^212 ^Pb/^212^Bi generator and the ^224^Ra/^212 ^Pb/^212^Bi generator [12, 13, 14, 15, 16, 17, 18]. However, regardless of whether ^228^Th or ^224^Ra is used as the “cow”, both face an unavoidable problem: where does the ^228^Th or ^224^Ra come from? These two nuclides are also extremely scarce at present. Another feasible approach to obtain ^212^Pb/^212^Bi is to directly and selectively extract these two nuclides from the decay chain of natural ^232^Th [10], as shown in Figure 1. Theoretically, 1000 t of ^232^Th can produce approximately 100 Ci of ^212^Pb/^212^Bi via decay in ∼20 years [19]. Without changing the decay equilibrium of ^232^Th–^228^Ra–^228^Ac–^228^Th–^224^Ra, the generation of ^212^Pb/^212^Bi occurs quickly, and these isotopes can be extracted repeatedly every 2–3 days [20]. If the technology for directly extracting ^212^Pb/^212^Bi from ^232^Th could be established, the cost of ^212^Pb/^212^Bi is expected to be significantly reduced. The 10.6 h half‐life of ^212^Pb also makes it possible to transport without relying on generators. Ideally, ^212^Pb/^212^Bi supply bases could be established in different locations to meet the research and application needs of ^212^Pb/^212^Bi in different provinces and cities. The half‐life of ^212^Bi is 60.55 min, allowing it to be utilized locally.

The selective extraction of ^212^Pb from the ^232^Th decay chain is highly challenging. Even under a long‐term equilibrium state, the mass ratio of ^212^Pb to ^232^Th is astonishingly low at 1:10^13^, making the separation of ^212^Pb extremely difficult. To date, reports on this topic are limited to four publications, with three focusing on special supramolecular recognition materials to achieve the selective separation of ^212^Pb from the ^232^Th decay chain [10, 20, 21, 22]. In 2022, Chen et al. [20] at Peking University first reported the direct extraction of 2.2 MBq of ^212^Pb from 2.5 kg of thorium nitrate using a commercially available Pb‐resin loaded with 4',4''(5'')‐di‐tert‐butyl‐18‐crown‐6 (DtBuDC18C6), with a chemical recovery rate of approximately 83% and a radionuclide purity of 99.9%. Subsequently, in 2024, Cao. et al. [21] reported the synthesis of a 2,13‐disulfonic acid diethylamino dibenz‐18‐crown‐6 (DSADB18C6) extractant by introducing sulfonic acid groups and grafting hydrophobic alkyl chains onto the dibenz‐18‐crown‐6 (DB18C6) skeleton, and achieved the separation of trace amounts of ^212^Pb from the ^232^Th decay chain through liquid–liquid extraction, with a chemical recovery rate of approximately 85.7%. In 2025, Cao et al. [22] reported again that by combining the DtBuDC18C6 reagent with cloud point extraction, they achieved the separation of ^212^Pb from the ^232^Th decay chain with a chemical recovery rate of approximately 53.8%. Although the above three reports demonstrate initial success in the selective separation of ^212^Pb from the decay chain of ^232^Th, several key challenges remain. First, the extraction of ^212^ Pb from thorium via liquid–liquid extraction not only requires large amounts of organic solvents and extractants, but also generates a large amount of radioactive waste liquid. Second, crown ethers cannot achieve absolute selectivity in extracting Pb(II); they also have a weak extraction capacity for Th(IV) [21, 22], leading to a contamination to the ^212^Pb product. Third, the synthesis of crown ethers is difficult, and their price is comparable to that of gold, resulting in a high extraction cost for ^212^Pb. Fourth, crown ethers have significant water solubility, with a saturation solubility of hundreds of ppm [22]. As a result, when used for the extraction of medical isotopes, the crown ethers can be lost with the feed of solution and cause organic pollution.

In 2024, we reported a case of directly and selectively extracting ^212^Pb from the ^232^Th decay chain without the use of crown ethers as extractant [10]. We adopted a completely different method based on a low‐cost adsorption material. In that work [10], we demonstrated that a silica‐based anion exchange resin grafted with 1‐methylpyridyl groups could realize the direct extraction of Pb(II) and Bi(III) in HCl medium; other elements in the simulated ^232^Th decay chain, including Th(IV), La(III) (simulating Ac(III)), and Ba(II) (simulating Ra(II)), were not adsorbed. Based on the above finding, we achieved the simultaneous separation of ^212^Pb and ^212^Bi from 2 g Th(NO_3_)_4_·*x*H_2_O without the use of crown ethers for the first time.

Although our work described above represents significant progress, the SiPyR‐N4 resin showed a relatively low affinity for the key element Pb in HCl medium, with an adsorption efficiency of only ∼5% [10]. As a result, Pb(II) was prone to leakage during the dynamic adsorption by the column. Moreover, the isolated ^212^Pb and ^212^Bi were not subjected to quantitative analysis in that work, leading to the failure of calculation on chemical recovery rate. After 1 year of building on that work, we report in this paper a significantly improved, practically valuable strategy and principle for the ^212^Pb/^212^Bi extraction from the ^232^Th and its daughters. Using this novel strategy, we successfully extracted 2.07 MBq of ^212^Pb from 2 kg Th(NO_3_)_4_·*x*H_2_O, of which the radionuclide purity exceeded 99.9%, and the chemical recovery rate reached a record‐breaking 88.2%. To date, this work represents the first report of successfully extracting ^212^Pb/^212^Bi from ^232^Th and its daughters using anion exchanger in bromide medium, as well as the mechanism illustrations. It perfectly solved all the defects of crown ethers in the extraction of ^212^Pb from the ^232^Th decay chain, and enables the supply of ^212^Pb/^212^Bi directly from the abundant natural thorium resource to be possible, thus having significant theoretical and practical value.

## 2 Results and Discussion

### 2.1 Static Adsorption of Pb(II) and Bi(III) on SiPyR‐N4 in Different Media

**FIGURE 2:**
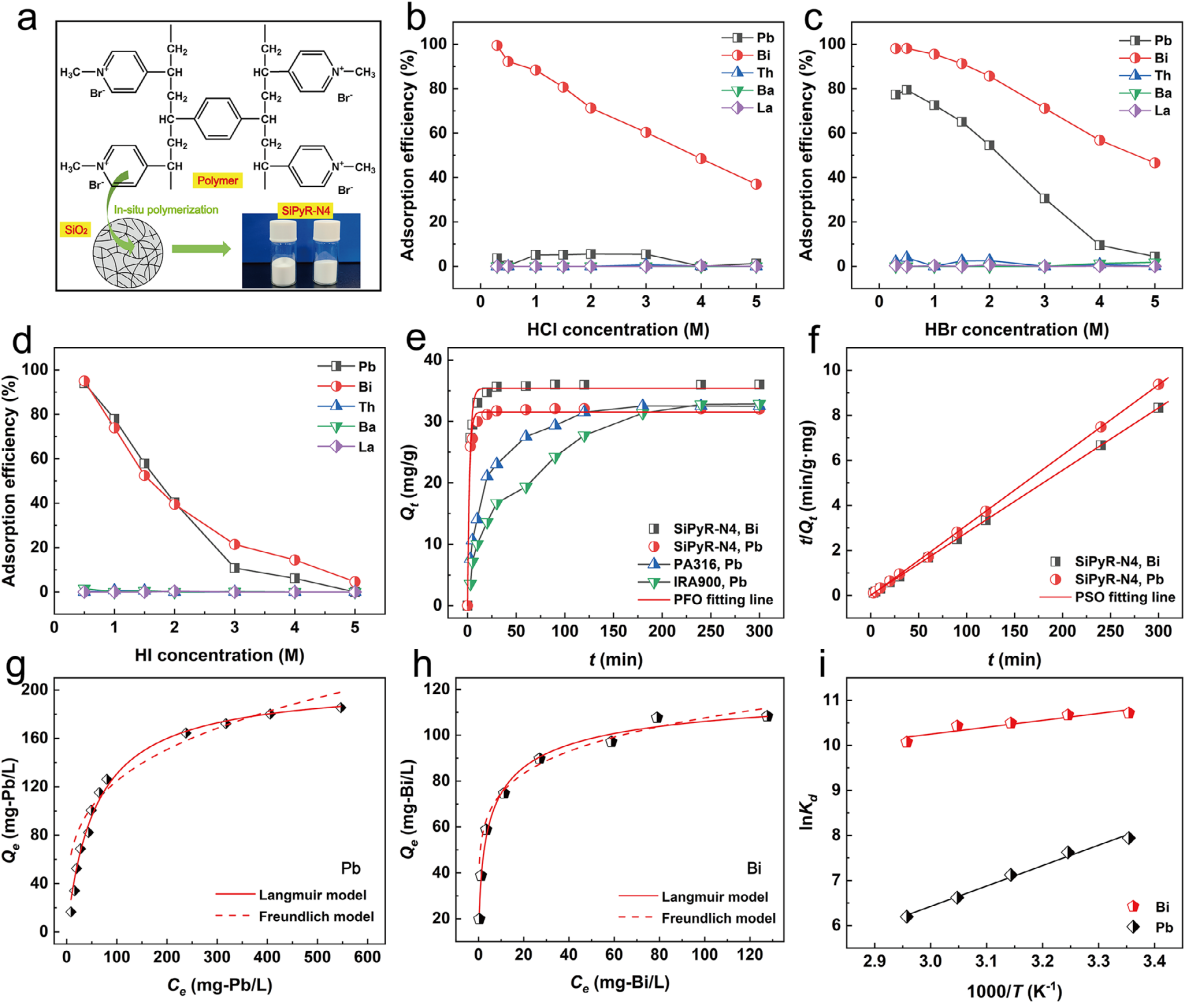
Adsorption behaviors of SiPyR‐N4 for Pb(II) and Bi(III). a) Structure and appearance of SiPyR‐N4. b) Adsorption of different elements by SiPyR‐N4 in HCl solution. c) Adsorption of different elements by SiPyR‐N4 in HBr solution. d) Adsorption of different elements by SiPyR‐N4 in HI solution. e) Adsorption kinetics of SiPyR‐N4 fitted by the pseudo‐first‐order (PFO) kinetic model. f) Adsorption kinetics of SiPyR‐N4 fitted by the pseudo‐second‐order (PSO) kinetic model. g) Adsorption isotherm and model fitting of SiPyR‐N4 for Pb(II). h) Adsorption isotherm and model fitting of SiPyR‐N4 for Bi(III). i) Adsorption thermodynamics and model fitting of SiPyR‐N4 for Pb(II) and Bi(III).

Here, we used the previously prepared SiPyR‐N4 resin, for which the preparation and characterization are reported in our previous works [10]. SiPyR‐N4 is a porous silica‐based anion exchange resin that exhibits extremely fast adsorption/desorption kinetics and a low column pressure drop. Such characteristics makes it highly suitable for the rapid extraction of nuclides with short half‐lives. The structures of the organic polymer and silica in the resin along with the resin appearance are shown in Figure 2a.

Compared with our previous work in 2024 [10], the selective affinity of SiPyR‐N4 for Pb(II) in the simulated ^232^Th decay chain was significantly improved after replacing the HCl medium with HBr or HI medium; the maximum adsorption efficiency of Pb(II) increased from 5.4% (HCl) to 79% (HBr) and 94% (HI), as shown in Figure 2b–d. Meanwhile, SiPyR‐N4 maintained a high adsorption efficiency for Bi(III), with basically no adsorption of Th(IV), Ba(II), or La(III). These results suggest that HBr and HI are more suitable extraction media than HCl. An adsorption efficiency of 79% (HBr) or 94% (HI) indicates that Pb(II) in solution will be immobilized tightly by the resin column without leakage. Considering the instability of HI solution in atmosphere, we finally selected HBr as the extraction medium for subsequent experiments, and 0.5 m was determined as the optimal concentration based on the experimental results.

Next, we systematically investigated the adsorption behaviors of SiPyR‐N4 by analyzing the adsorption kinetics, isotherms, and thermodynamics for Pb(II) and Bi(III) in 0.5 m HBr solution. As shown in Figure 2e, SiPyR‐N4 shows extremely fast adsorption speed, with adsorption equilibrium reached within 20 min for Pb(II) and 30 min for Bi(III). For comparison, the commercial anion exchange resins PA316 and IRA900 required 180 and 240 min, respectively, to reach adsorption equilibrium for Pb(II) under the same conditions. In terms of kinetics, SiPyR‐N4 was more over nine folds faster than the commercial resins. Classical kinetic models were then used to fit the kinetic data. Based on the *R* ^2^ values obtained for the model fits, the PSO model better described the kinetic data than the PFO model (Figure 2e,f), indicating that the capture of Pb(II) and Bi(III) were both controlled by chemical reactions [23, 24]. The fitting parameters are provided in Table S1.

Next, the effects of the equilibrium concentrations of Pb(II) and Bi(III) on the adsorption amounts were evaluated. The adsorption isotherms are shown in Figure 2g,h. As the equilibrium concentrations increased, the adsorption amounts for both Pb(II) and Bi(III) first increased quickly and then remained constant after reaching plateaus. The maximum adsorption amounts for Pb(II) and Bi(III) on SiPyR‐N4 were experimentally determined to be 185 and 108 mg/g, respectively, equivalent to 0.89 and 0.52 mmol/g, respectively. The Langmuir and Freundlich models [19, 25] were used to fit the adsorption isotherms. According to the correlation coefficients *R* ^2^, the Langmuir model provided the best fit to the adsorption data, suggesting that the adsorption of Pb(II) and Bi(III) by SiPyR‐N4 both followed monolayer adsorption mechanisms [25]. The corresponding fitting parameters are shown in the Tables S2 and S3.

The adsorption thermodynamics for Pb(II) and Bi(III) were investigated by varying the ambient temperature. As shown in Figure 2i, the distribution coefficients *K_d_* of SiPyR‐N4 for Pb(II) and Bi(III) both decreased with increasing temperature, and we observed linear relationships between ln*K_d_* and 1/*T*. These data were further fitted using the Arrhenius equations [10], and then the changes in enthalpy Δ*H* and Gibbs free energy Δ*G* for the adsorption reactions of Pb(II) and Bi(III) were calculated based on the slope and intercept of the fitted line. The negative values of Δ*H* and Δ*G* (Table S4) demonstrate that both adsorption processes were spontaneous exothermic reactions.

### 2.2 Dynamic Adsorption and Separation of Pb(II) and Bi(III)

**FIGURE 3:**
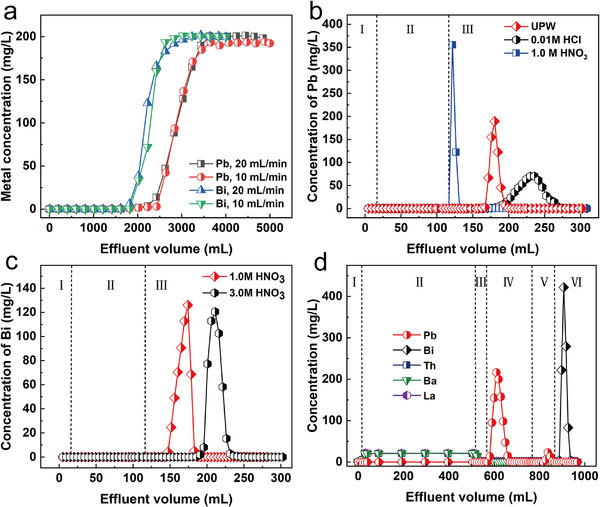
Pb/Bi separation from simulated ^232^Th decay chains. a) Breakthrough curves of Pb(II) and Bi(III) under different flow rates. b) Desorption curves for Pb(II) using different eluants (I: dead volume, II: 25 mg/L Pb + 0.5 m HBr, III: eluant). c) Desorption curves for Bi(III) using different eluants (I: dead volume, II: 25 mg/L Bi + 0.5 m HBr, III: eluant). d) Pb and Bi separation from the simulated decay chain of ^232^Th (I: dead volume, II: 20 mg/L Pb + 20 mg/L Bi + 20 mg/L Th + 20 mg/L Ba + 20 mg/L La + 0.5 m HBr, III: 0.5 m HBr, IV: UPW, V: 0.1 m HCl, VI: 1.0 m HNO_3_).

To realize the efficient extraction of Pb(II) and Bi(III) as much as possible, the dynamic adsorption and elution of Pb(II) and Bi(III) by SiPyR‐N4 were further investigated in column. Figure 3a shows the breakthrough curves of Pb(II) and Bi(III) in the SiPyR‐N4 resin column, which both presented typical S shapes. During the experiment, approximately 1820 mL of Pb solution and 2430 mL of Bi solution could be fixed by the column without leakage, corresponding to approximately 230 B.V. (bed volume) and 310 B.V., respectively. Moreover, the same immobilization performances were maintained at a high flow rate of 20 mL/min (equivalent to 2.5 B.V./min) as at a low flow rate of 10 mL/min (equivalent to 1.3 B.V./min). This demonstrates the excellent column separation performance of SiPyR‐N4 and suggests that it can be applied to the treatment of fluids under high flow rates, which is beneficial for the separation of nuclides with short half‐lives.

Figure 3b,c shows the elution curves of Pb(II) and Bi(III) with different eluants. As shown in Figure 3b, efficient Pb(II) elusion was achieved in both 1.0 m HNO_3_ and ultra‐pure water (UPW). In contrast, the elution effect of 0.01 m HCl was relatively poor, as manifested by the wide elution peak in Figure 3b. As shown in Figure 3c, the Bi(III) elution effect of 1.0 m HNO_3_ was better than those of higher concentrations of HNO_3_ (e.g., 3.0 m). Based on the above experimental results, we used UPW and 1 m HNO_3_ as the eluants for Pb(II) and Bi(III), respectively, and successfully achieved the simultaneous separation of Pb and Bi from the simulated decay chain of thorium (Figure 3d).

### 2.3 Scaled‐Up Cool and Hot Separation Verification

**FIGURE 4:**
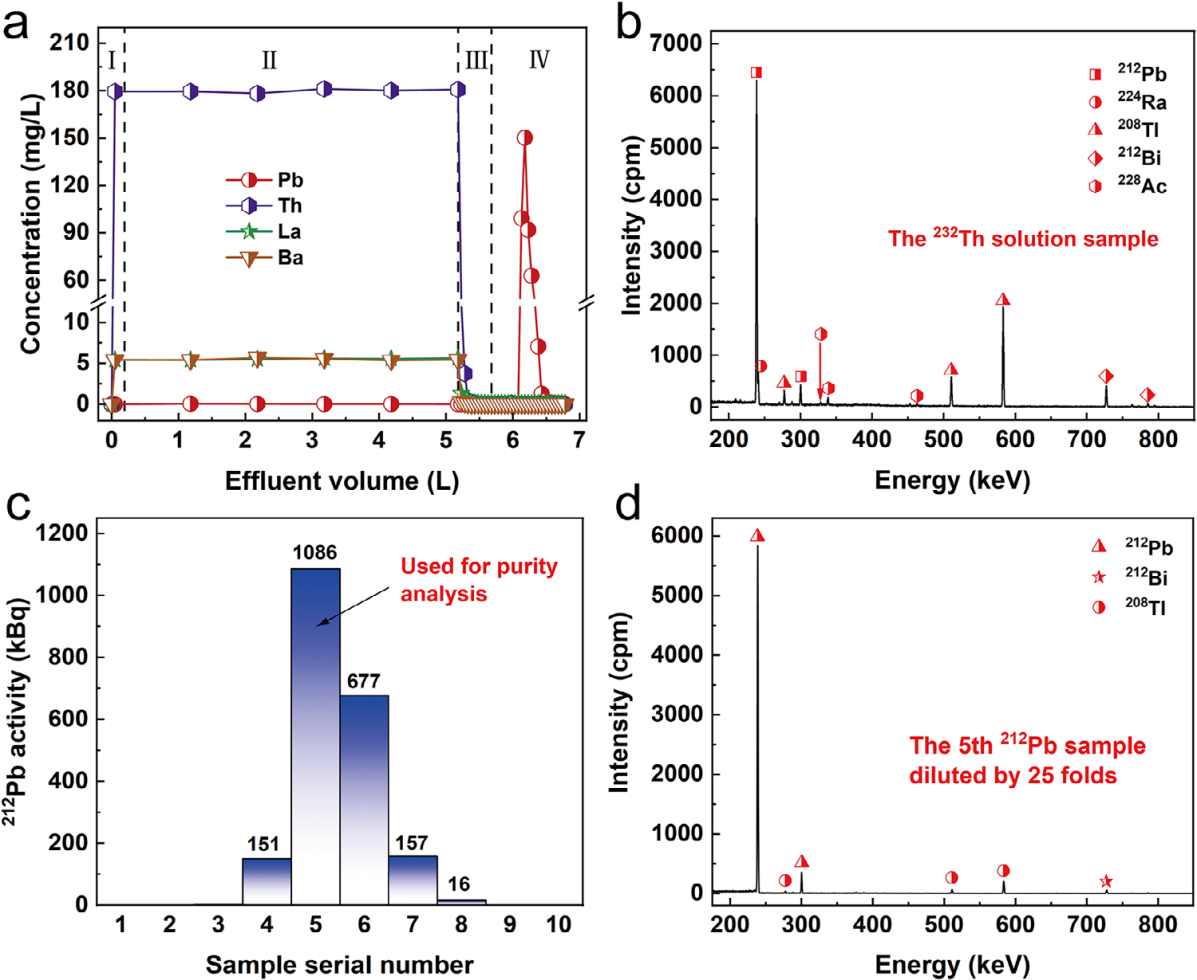
Scaled‐up separation of Pb from the simulated and real decay chain of ^232^Th: a) Pb separation from the simulated decay chain of ^232^Th (I: dead volume, II: 5 mg/L Pb(II) + 200 mg/L Th(IV) + 5 mg/L Ba(II) + 5 mg/L La(III) + 0.5 m HBr, III: 0.5 m HBr, IV: 1.0 m HNO_3_ as eluant). b) Gamma spectrum of the source solution containing ^232^Th. c) ^212^Pb elution results after the feed of 5 L thorium solution with high concentration (each serial number represents 100 mL of eluant). d) Gamma spectrum of the fifth eluant sample.

Considering that 1.0 m HNO_3_ showed better elution performance for Pb(II) compared to UPW, and the half‐life of ^212^Bi (T_1/2_ = 60.5 min) is too short, we finally decided to use 1.0 m HNO_3_ as the eluant in a scaled‐up experiment for the separation of ^212^ Pb. The results of the cool separation experiment using stable nuclides as surrogates are shown in Figure 4a. During the adsorption stage, Th(IV), Ba(II), and La(III) were not immobilized by the resin column and passed through directly. In contrast, Pb(II) was retained in the column. After introducing a certain amount of rinsing reagent and eluant, Pb(II) was completely desorbed from the resin column and was separated thoroughly from Th(IV), Ba(II), and La(III), with the chemical recovery rate reaching 100%.

Based on the results of the cool separation experiment, we performed a hot separation experiment using 2 kg of Th(NO_3_)_4_·xH_2_O as the source and the same method as the cool experiment. The separation results are shown in Figure 4b–d. Before processing, ^224^Ra, ^228^Ac, ^212^Pb, ^212^Bi, and ^208^Tl were clearly observed in the gamma spectrum of ^232^Th solution along with some interfering peaks involving other nuclides. The amount of ^212^Pb in 2 kg of Th(NO_3_)_4_·xH_2_O was determined to be 2.38 MBq by gamma spectrometry. After separation in our column, the ^212^Pb in the thorium solution was successfully isolated; the eluted ^212^Pb was mainly distributed in the fourth, fifth, sixth, and seventh eluant samples, with a total activity of 2.07 MBq. The chemical yield of ^212^ Pb reached a record‐breaking value of 88.2%. In the ^212^Pb sample, no other impurities such as ^224^Ra and ^228^Ac were detected, and the peak intensities of its daughter nuclides ^208^Tl and ^212^Bi were also low. These results demonstrate that the obtained ^212^ Pb product had a high radionuclide purity, estimated to exceed 99.9%. The on‐site experimental devices and experimenters for the hot separation are shown in Figure S1.

### 2.4 Mechanism Analysis

**FIGURE 5:**
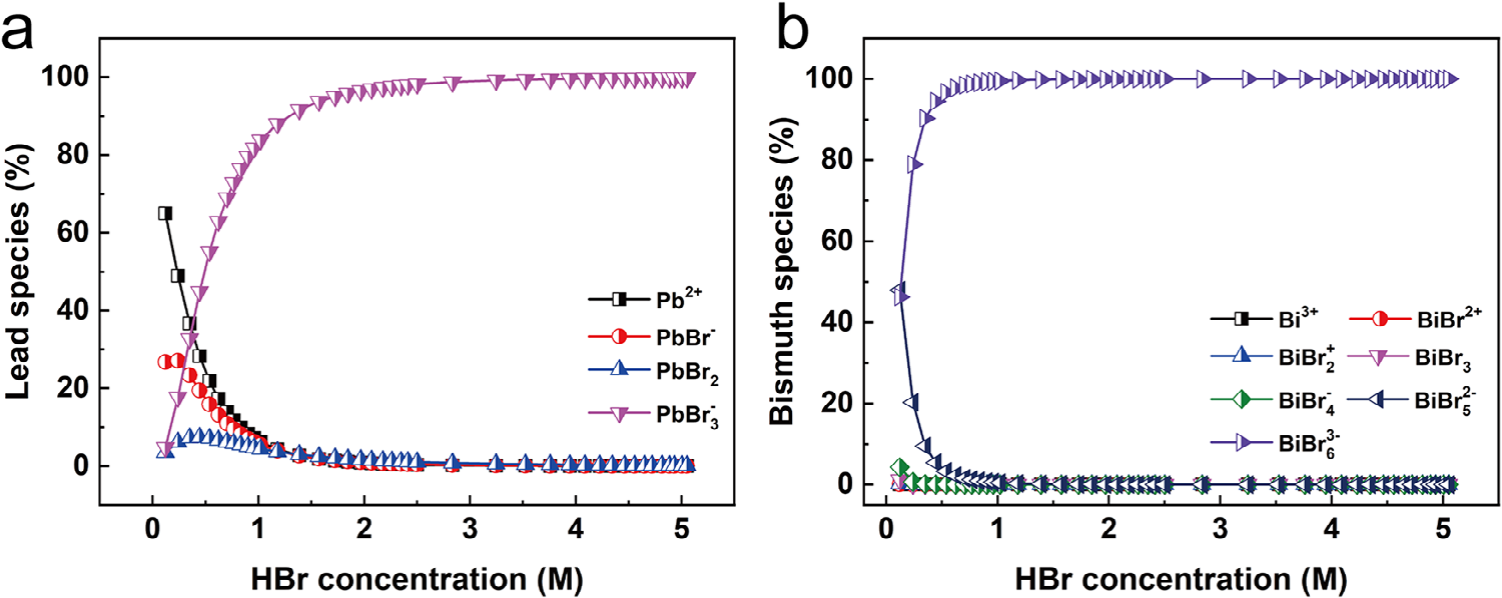
Chemical species distribution of different metal cations in HBr medium. a) Pb(II). b) Bi(III).

To better understand the adsorption behavior of SiPyR‐N4, we calculated the species distributions of Pb(II) and Bi(III) in different concentrations of HBr solution based on their thermodynamic stability constants (Figure 5a,b) [26, 27]. In 0.5 m HBr, four chemical species of Pb(II) were predicted: Pb^2+^ (21.8%), PbBr^+^ (15.9%), PbBr_2_ (7.1%), and PbBr_3_^−^ (55.2%). Meanwhile, seven species of Bi(III) were predicted: Bi^3+^ (∼0%), [BiBr]^2+^ (∼0%), [BiBr_2_]^+^ (∼0%), [BiBr_3_] (∼0%), [BiBr_4_]^−^ (0.03%), [BiBr_5_]^2−^ (3.3%), and [BiBr_6_]^3−^ (96.7%). As the HBr concentration increased, the proportions of [PbBr_3_]^−^ and [BiBr_6_]^3−^ both increased continuously until reaching their maximum values near 100%. Apparently, a high concentration of HBr promotes the formation of Pb and Bi anion complexes; however, the high concentration of Br^−^ will also compete with the Pb(II) and Bi(III) anion complexes for active sites, which explains the decreases in Pb(II) and Bi(III) adsorption efficiencies with increasing HBr concentration (Figure 2b).

**FIGURE 6:**
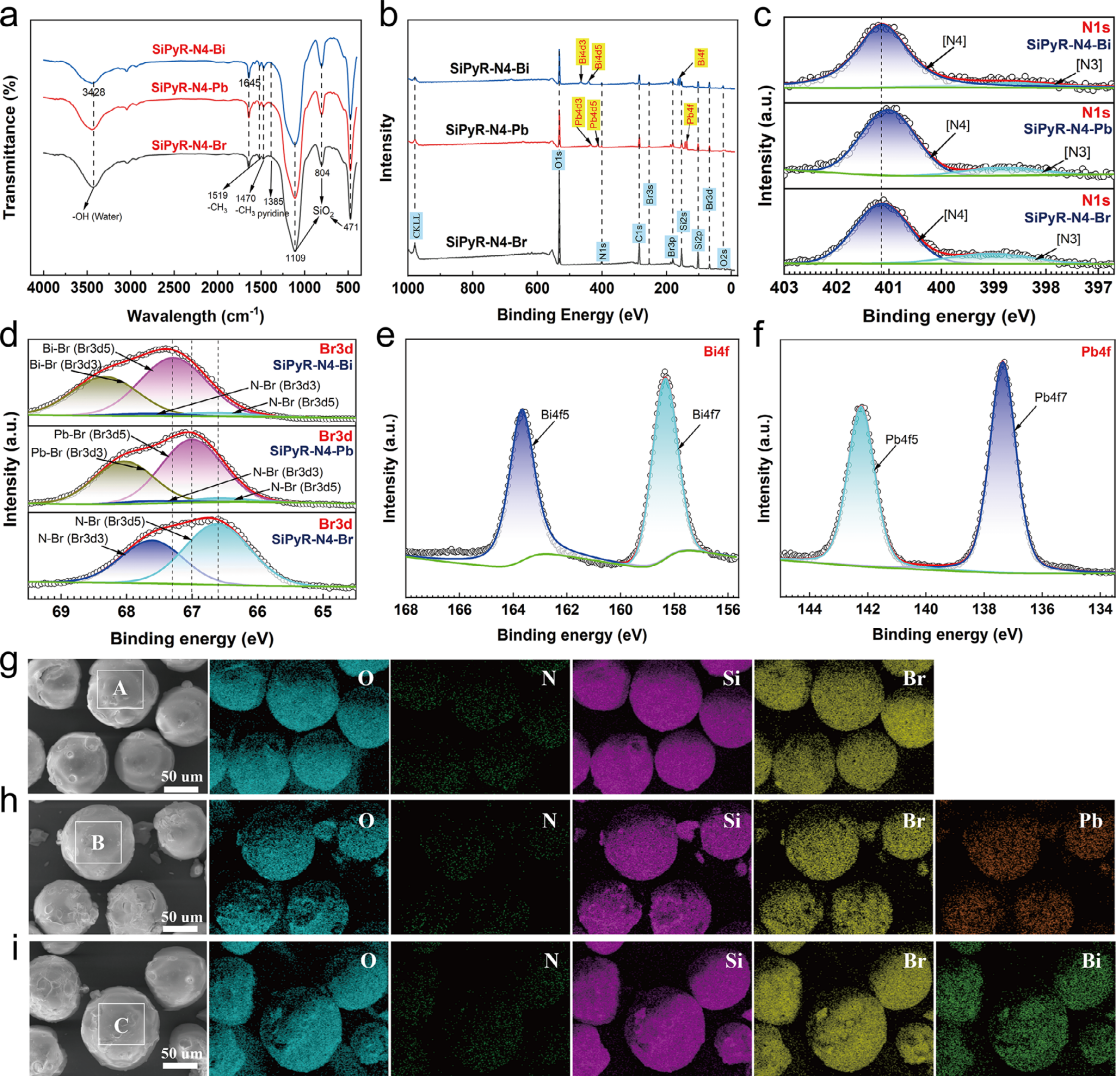
Characterization of SiPyR‐N4 before and after Pb(II) and Bi(III) adsorption. a) FT‐IR spectra. b) XPS full‐scan spectra. c) XPS fine spectra of N1s. d) XPS fine spectra of Br3d. e) XPS fine spectra of Pb4f on SiPyR‐N4‐Pb. f) XPS fine spectra of Bi4f on SiPyR‐N4‐Bi. g–i) SEM‐EDS images of SiPyR‐N4‐Br, SiPyR‐N4‐Pb and SiPyR‐N4‐Bi.

To provide more information about the adsorption mechanisms, SiPyR‐N4 samples before (denoted as SiPyR‐N4‐Br) and after the adsorption of Pb (denoted as SiPyR‐N4‐Pb) and Bi (denoted as SiPyR‐N4‐Bi) were analyzed by Fourier‐transform infrared spectroscopy (FT‐IR) and X‐ray photoelectron spectroscopy (XPS). As shown in Figure 6a, due to the masking of the strong characteristic peaks of SiO_2_ (471, 804, and 1109 cm^−1^), only the characteristic peaks belonging to pyridine (1385 cm^−1^) and methyl (1470 and 1519 cm^−1^) were found in the FT‐IR spectrum [10]. Almost no other information was detected on the adsorption of Pb(II) and Bi(III). However, in the XPS full spectrum of SiPyR‐N4‐Pb and SiPyR‐N4‐Bi (Figure 6b), characteristic peaks belonging to Pb and Bi were clearly observed, confirming that Pb(II) and Bi(III) were indeed adsorbed by the resin. Figure 6c suggests that adsorption of Pb(II) or Bi(III) resulted in a nearly negligible change in the binding energy of N1s electrons, indicating that Pb(II) and Bi(III) did not directly form chemical bonds with N atoms; rather, Pb(II) and Bi(III) might have connected to N atoms through Br bridges, resulting in almost no change in the binding energy of N1s electrons during adsorption. Figure 6d shows the fine Br3d XPS spectra. For SiPyR‐N4‐Br, the two peaks at 66.6 and 67.6 eV can be attributed to Br3d5 and Br3d3, respectively, but both belong to N─Br. However, after the adsorption of Pb(II) or Bi(III), two new peaks belonging to Pb─Br (at 67.0 and 68.0 eV) or Bi─Br (at 67.3 and 68.3 eV) appeared, and the peaks belonging to N─Br nearly disappeared. These changes indicate that Pb(II) and Bi(III) were indeed adsorbed in the form of Pb─Br complexes and Bi─Br complexes, respectively. The fine XPS spectra of Pb4f and Bi4f (Figure 6e,f) do not show signals of Pb or Bi in different valence states, demonstrating that no redox reaction occurred.

Scanning electron microscopy (SEM) with energy‐dispersive X‐ray spectroscopy (EDS) was further conducted on SiPyR‐N4‐Br, SiPyR‐N4‐Pb and SiPyR‐N4‐Bi. As shown in Figure 6g–i, different elements were uniformly distributed on the surface of the resins, such as Br, Si, N and O. By further scanning region A on SiPyR‐N4‐Br, region B on SiPyR‐N4‐Pb, and region C on SiPyR‐N4‐Bi, we obtained the atomic ratios of different elements, as shown in Table S5 and Figure S2. In region A, the atomic ratio of Br to N was approximately 0.959:1. In regions B and C, the Br:N atomic ratios were 2.38:1 and 1.75:1, respectively. The increases in the Br/N atomic ratio confirm that Pb(II) and Bi(III) were adsorbed in the form of Br‐containing complexes by the resin, consistent with the results of XPS characterization. In regions B and C, the Pb:Br and Bi:Br atomic ratios were 3.03:1 and 5.26:1, respectively. SiPyR‐N4‐Pb and SiPyR‐N4‐Bi were prepared in the presence of excess Pb(II) and Bi(III); if all the Br^−^ carried by the SiPyR‐N4 resin participated in coordination with Pb(II) and Bi(III), the Pb:Br atomic ratio of 3.03:1 corresponds exactly to the [PbBr_3_]^−^ complex, while the Br:Bi atomic ratio of 5.26:1 reflects both the [BiBr_5_]^2−^ and [BiBr_6_]^3−^ complexes. As discussed earlier, the adsorption data were well fitted by the PSO model and the Langmuir model, indicated that the immobilization of Pb(II) and Bi(III) occurred via chemical reaction and monolayer adsorption. Therefore, according to the Br:Bi atomic ratio, the molar ratio of [BiBr_5_]^2−^ to [BiBr_6_]^3−^ adsorbed can be calculated as approximately 2.85:1. Thus, [BiBr_5_]^2−^ apparently occupied a dominant position. Although this result differs from the calculated proportions of Bi(III) species, it is still reasonable because different species can be transformed during the adsorption reaction [28].

Based on the above results, we further determined the total exchange capacity of SiPyR‐N4‐Br to be 1.15 meq/g through titration and compared it with the maximum adsorption capacities of SiPyR‐N4 for Pb(II) and Bi(III) in 0.5 m HBr solution. The observed maximum adsorption capacities of SiPyR‐N4 for Pb(II) and Bi(III) were 0.89 mmol‐Pb/g and 0.52 mmol‐Bi/g, respectively, as shown in Figure 2g,h. In fact, the saturation value was difficult to observe, and the ratios of the total exchange capacity to the above two observed values were 1.29:1 and 2.21:1. From these two measured ratios, we speculate that the Pb(II) species adsorbed by the resin mainly carried one unit of negative charge, while the adsorbed Bi(III) species mainly carried two units of negative charge. These results are basically consistent with the atomic ratios determined from SEM‐EDS analysis. Hence, we confirmed that the Pb(II) and Bi(III) species adsorbed by the resin were primarily [PbBr_3_]^−^ and [BiBir_5_]^2−^. Simultaneously, some [BiBr_6_]^3−^ may have also been adsorbed by the resin.

### 2.5 Density Functional Theory Analysis

**FIGURE 7:**
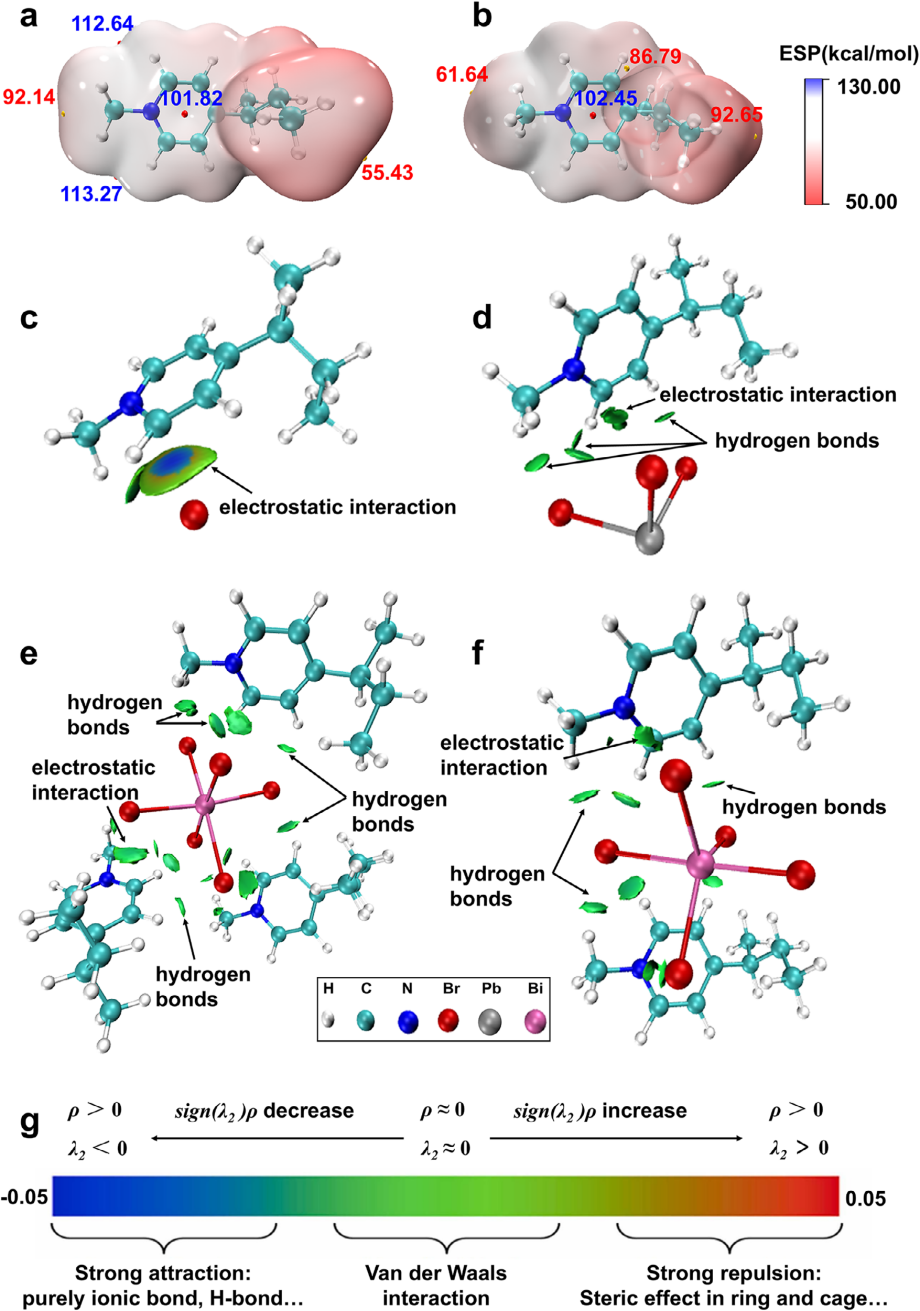
Electronic structure analysis of MBP^+^ cation and its interactions with anions. a, b) ESP maps of the isolated MBP^+^ cation viewed from two different orientations, highlighting its cationic character and potential anion‐binding sites. c–f) IGMH isosurfaces (isovalue = 0.01 a.u.) visualizing the specific interactions within the complexes formed between MBP^+^ and Br^−^, PbBr_3_^−^, BiBr_6_^3−^, and BiBr_5_^2−^. g) Standard color scheme for interpreting the sign(λ_2_)ρ function on the IGMH isosurfaces.

We conducted DFT calculations to obtain more information on the mode of interaction between SiPyR‐N4 and the Pb(II) or Bi(III) anion complexes. Here, the cationic resin was simplified using its minimal structural unit: 1‐methyl‐4‐sec‐butyl pyridine (abbreviated as MBP^+^). The optimized structures of MBP^+^ and its complexes with various anions, along with other relevant configurations, are provided in Figures S3 and S4. The electrostatic potential (ESP) surfaces of the cationic resin MBP^+^, which visually depict alterations in its charged regions, are shown from two different perspectives in Figure 7a,b. As expected for a cationic species, the ESP values across the molecular surface were uniformly positive. Notably, the cavity formed by the pyridine ring and the longer alkyl chain of the sec‐butyl group (Figure 7a) exhibits markedly higher positive potentials (indicated by blue labels) compared to the other regions (Figure 7b), suggesting a preferred binding site for anion adsorption. In aqueous HBr solution, this active region facilitated the adsorption of Br^−^ ions, leading to the formation of a stable bromide complex, MBPBr. The associated ΔG for this process reaction (MBP^+^ + Br^−^ → MBPBr) is −32.0 kcal/mol (Table S6), indicating that the adsorption process occurred spontaneously. Thus, in HBr aqueous solution, the cationic resin predominantly existed in the bromide form, MBPBr. Additionally, from a structural stability perspective, the lowest energy configuration of MBPBr (MBPBr‐I in Figure S3) supports that the region identified in Figure 7a is the optimal active site for anion adsorption on the cationic resin.

In aqueous solutions of HBr containing metal ions such as Th^4+^, Ac^3+^, Pb^2+^, and Bi^3+^, their speciation depends on the hard‐soft acid‐base (HSAB) principle [29, 30]. As typical hard acids, Th^4+^ and Ac^3+^ exhibit much stronger affinity for the hard base H_2_O than for the soft base Br^−^. Therefore, Th^4+^ and Ac^3+^ predominantly exist as hydrated ions like [Th(H_2_O)_8_]^4+^ and [Ac(H_2_O)_9_]^3+^ [31, 32]. For instance, for [Th(H_2_O)_8_]^4+^, the ΔG value of further water coordination to form [Th(H_2_O)_9_]^4+^ (ΔG = −9.79 kcal/mol) is more negative than that of ligand exchange with Br^−^ to form [Th(H_2_O)_7_Br]^3+^ (ΔG = −6.14 kcal/mol; Table S7). This indicates that Br^−^ ions are unlikely to replace the inner‐sphere water molecules of Th^4+^, consistent with the HSAB principle. In contrast, the borderline acids Pb^2+^ and Bi^3+^ coordinate more strongly with Br^−^ than with H_2_O. Pb^2+^ has been shown to form highly stable PbBr_3_^−^ complexes in similar systems [26]. Based on the literature and our experimental results, we evaluated the thermodynamic stability of PbBr_3_^−^, BiBr_5_^2−^, and BiBr_6_^3−^ complexes. As shown in Table S7, the ΔG values for the relevant reactions range from −72.85 to −239.71 kcal/mol, confirming the spontaneous formation and high thermodynamic stability of these bromo‐complex anions in HBr solution. Thus, the fundamental differences in the speciation of metal ions in HBr solution allowed the brominated cationic resin MBPBr to selectively adsorb Pb and Bi bromo‐anions via anion exchange. Meanwhile, Th(IV) and Ac(III) were not adsorbed, enabling the highly efficient and selective separation of Pb(II) and Bi(III). Table S6 further presents the ion exchange reactions between MBPBr and the PbBr_3_^−^, BiBr_5_^2−^, and BiBr_6_^3−^ anions along with the corresponding changes in Gibbs free energy (−10.78 to −29.37 kcal/mol), confirming the spontaneity of the adsorption process.

To intuitively visualize the chemical bonds and weak interactions between molecular fragments, we employed the independent gradient model based on Hirshfeld partition (IGMH) analysis with MBP^+^ cation and the bromo‐anions as distinct fragments (Figure 7c–g). The blue isosurfaces represent strong attractive interactions, whereas green regions indicate weak interactions such as van der Waals forces. In MBPBr, the resin cation primarily interacts with the Br^−^ anion via electrostatic attraction (Figure 7c). For the complexes formed between the resin cation and Pb/Bi bromo‐anions, both electrostatic interactions and hydrogen bonding play important roles. As shown in Figure 7d–f, electrostatic and van der Waals interactions occur between the anions and the pyridinium ring plane, while C─H─Br hydrogen bonds are formed between the H atoms on the pyridinium ring or alkyl chains and the Br atoms of the anions. Both of these interactions contribute significantly to the stability of the complexes, as reflected by their lowest‐energy configurations (Figure S3).

It is worth noting that although performing DFT calculations can provide useful insights, the simplified MBP model employed in the present calculations still has difference from the actual cross‐linked polymer resin environment. Consequently, the DFT calculations reported herein are subject to inherent limitations, and the resulting insights should be interpreted as a qualitative description of the interaction mechanism rather than a quantitative assessment.

### 2.6 Material Reusability

**FIGURE 8:**
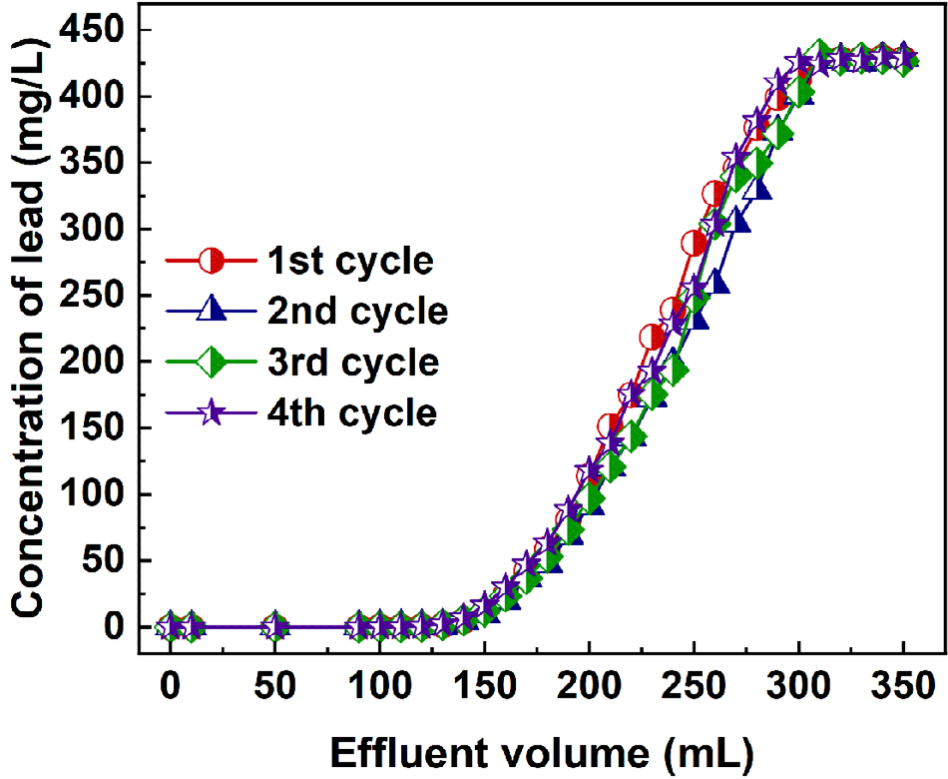
Change of the breakthrough curves for SiPyR‐N4 with the increasing rounds of adsorption–desorption cycle.

The stability and recyclability of the adsorption materials are crucial for potential practical applications, and these performances for SiPyR‐N4 were evaluated by repeating the adsorption‐desorption cycle in column. As shown in Figure 8, the breakthrough curves for all four cycles exhibit nearly identical profiles, indicating that both adsorption capacity and kinetics remained essentially unchanged upon repeated adsorption–desorption cycling. These results demonstrate the exceptional chemical and mechanical stability of the SiPyR‐N4 resin, as well as its robust recyclability under operational conditions.

### 2.7 Comparison to Existing Method for ^212^ Pb Acquisition

This study introduces an advanced methodology and functional material for the direct, selective separation of ^212^Pb/^212^Bi from the ^232^Th decay chain. Using the SiPyR‐N4 resin, only ^212^ Pb and ^212^Bi are selectively adsorbed, while ^232,228^Th, ^228,224^Ra, and ^228^Ac remain quantitatively in the aqueous phase, with their radioactive activities fully retained. As a result, the extracted ^212^ Pb/^212^Bi can be rapidly regenerated within 2–3 days.

Compared to conventional ^228^Th/^224^Ra/^212 ^Pb/^212^Bi or ^224^Ra/^212 ^Pb/^212^Bi generator systems, this approach offers three principal advantages: (i) the widespread availability and low cost of ^232^Th as a starting material; (ii) the full reusability of the thorium‐bearing solution after ^212^Pb/^212^Bi extraction; and (iii) operational simplicity without intermediate radionuclide handling. Relative to crown ether–based ^212^Pb separation method, the SiPyR‐N4 resin has a substantially lower material cost and superior chemical stability. Cost analysis indicates that SiPyR‐N4 can be obtained at approximately CNY 2,000 per kilogram (shown in Section S4), whereas Pb‐resin is priced at CNY 546 000 per kilogram (provided by Beijing UDLER Technologies Co., Ltd., China) —representing a >260‐fold cost reduction. Moreover, crown ether ligands have high water solubility and are prone to leaching into aqueous media, compromising column integrity and radiopurity over repeated cycles. Collectively, the proposed method enables a long‐term, stable and low‐cost supply of ^212^Pb, demonstrating strong potential for industrial‐scale implementation. A comparative summary of key technical and economic parameters across representative ^212^Pb production strategies is provided in Table S8.

However, from an engineering perspective, this method still presents two important challenges. First, attaining a clinically relevant activity of ^212^Pb for a single human dose requires processing substantial volumes of ^232^Th solution per batch—leading to elevated radioactive inventory and correspondingly higher radiological risk compared with compact, shielded generator systems. Robust radiation safety protocols—including engineered shielding, remote handling infrastructure, and continuous dosimetry monitoring—are therefore indispensable to safeguard personnel and ensure regulatory adherence. Second, large‐scale ^212^Pb production is contingent upon the development and deployment of fully automated, GMP‐compatible manufacturing systems capable of delivering consistent yield, high radionuclide purity, and operational reproducibility.

## 3 Conclusion

We have, for the first time, demonstrated a highly promising method and material for selectively separating ^212^Pb and ^212^Bi from the decay chain of ^232^Th and clarified the mechanism of selective adsorption. Through the ingenious combination of coordination chemistry design and materials engineering, the extreme challenge of separating ultra‐trace target nuclides from ultra‐high concentration matrices was successfully overcome. Compared with our previous work, the selective adsorption efficiency of the silica‐based anion exchange resin for Pb(II) has been significantly improved, giving our present findings both significant theoretical significance and practical value. Using the novel method and anion exchanger, 2.07 MBq of ^212^Pb was successfully separated from ^232^Th and its daughters, with a record‐breaking chemical yield of 88.2% and an estimated radionuclide purity of over 99.9%. Mechanism analysis suggested that Pb(II) and Bi(III) could form anionic complexes in the HBr medium, with [PbBr^3^]^−^ and [BiBr_5_]^2−^ being the main species captured; while Th(IV), Ac(III) and Ra(II) mainly exist as cationic forms and thus were not fixed. To date, this represents the first report of successfully extracting ^212^Pb/^212^Bi from ^232^Th and its daughters using anion exchanger in bromide medium, as well as the mechanism illustrations. Meanwhile, it represents the second report worldwide of directly and selectively separating MBq‐level ^212^Pb from the decay chain of ^232^Th.

However, it is worth mentioning that the specific activity of the currently obtained ^212^Pb products remains relatively low, and residual bromide (Br^−^) — even at trace concentrations — poses a potential risk to their radiopharmaceutical utility. To address these challenges, we propose enhancing the current purification protocol by incorporating an additional cation‐exchange column, thereby establishing a two‐column separation‐enrichment process capable of simultaneously enriching ^212^Pb and achieving quantitative removal of Br^−^, and these will be performed in next work.

## 4 Experimental Section

### 4.1 Reagents and Materials

Th(NO_3_)_4_·xH_2_O (AR, 98%), PbCl_2_ (AR, 99.5%), Bi(NO_3_)_3_.5H_2_O (AR, 99%), La(NO_3_)_3_.6H_2_O (AR, 99.99%), Ba(NO_3_)_2_ (AR, 99.5%), concentrated HBr (40 wt.%), and HI (47 wt.%) were all purchased from Shanghai Macklin Biochemical Co., Ltd. (Shanghai, China). Th(NO_3_)_4_·xH_2_O (98%) that had been stored for a long time (also called old thorium) was provided by Hunan Zhonghe Jinyuan New Materials Co., Ltd. (Hengyang, China). Commercially available concentrated nitric acid and concentrated hydrochloric acid were provided by Guomao Chemical Reagent Co., Ltd. (Shanghai, China). All solutions were prepared using UPW with a resistivity of 18.2 mΩ. SiPyR‐N4 resin, which is a silica‐supported anion exchange resin carrying 1‐methylpyridine with Br^−^ as the contra ions, was prepared in the laboratory. The organic framework of SiPyR‐N4 is a copolymer of di‐vinylbenzene and 4‐ethylenepyridine, supported by an inorganic silica framework. The particle size of SiPyR‐N4 is 75∼150 µm, the BET specific surface area is approximately 52.2 m^2^/g, the pore volume is 0.54 mL/g, and the pore size is concentrated around 40 nm with an average value of 36.8 nm. The synthesis and characterization of SiPyR‐N4 were reported in detail in our recent work [10].

### 4.2 Batch Experiments

We conducted batch experiments to investigate the effects of acid type, acid concentration, contact time, equilibrium concentration, and temperature on the adsorption of Pb(II) and Bi(III) by SiPyR‐N4. The stock solution of thorium was prepared by dissolving a certain amount of Th(NO_3_)_4_·xH_2_O (AR, 98%) in UPW followed by the addition of a few drops of concentrated nitric acid for stabilization. The stock solutions of Ba(II), La(III), Pb(II), and Bi(III) were similarly prepared. The stock solutions containing the above metal ions were diluted with UPW and concentrated HBr to prepare a working solution containing five metal ions: Pb(II), Bi(III), Th(IV), Ba(II), and La(III). SiPyR‐N4‐Br (0.05 g) was mixed with 20 mL of working solution and placed in a water bath shaker at constant temperature for oscillation at 120 rpm. After 2 h, solid–liquid separation was performed using a microporous membrane filter, and the filtrate was diluted to an appropriate concentration for analysis by inductively coupled plasma atomic emission spectrometry (ICP‐AES; Ultima Expert, Horiba, Japan). Based on the changes in metal ion concentrations after adsorption, the adsorption rate and distribution coefficient of each metal ion were calculated. Each data point plotted in the figures represent the average values of two parallel samples, and the relative error was controlled within 5%. More details are described in Section S1.

### 4.3 Column Experiments

A glass column was fully filled with SiPyR‐N4‐Br to verify its dynamic adsorption performance. Screen plates were assembled at both ends of the column. The solution was pumped into the glass column through a pipeline by a peristaltic pump, and the effluent was collected at regular intervals using a fraction collector. After analyzing the average concentration of each metal ion in the effluent from different volume segments, the corresponding breakthrough curves and elution curves were plotted. Before beginning the experiment, bubbles in the column were removed using UPW, and enough HBr (0.5 m) was introduced to pre‐treat the glass column. seven different column experiments were performed, including ^212^Pb separation experiment and the dynamic lead adsorption‐desorption cycle experiment; the detailed experimental parameters of each column experiment are provided in Section S2. The concentration of each metal ion was determined by ICP‐AES, and the activities of radionuclides such as ^212^ Pb were measured using a gamma spectrometer equipped with a high‐purity germanium detector (GEM‐C45‐LB‐C, ORTEC, US).

### 4.4 Material Characterization

The SiPyR‐N4 resin was analyzed before and after adsorption by FT‐IR spectroscopy (IR Tracer 100, Shimadzu, Japan), XPS (ESCALAB Xi+, Thermo Scientific, USA), and SEM‐EDS (Tesscan Mira Lms, Czech) to provide information about the functional groups, atomic chemical environment, surface structure, and elemental distribution. The KBr pellet method was used to prepare the samples for FT‐IR spectroscopy. Pellets were prepared by mixing spectral‐grade KBr with the sample in a 100:1 ratio followed by grinding and pressing into a transparent sheet in a mold. For XPS analysis, XPSpeak software was used to separate overlapping peaks. For SEM‐EDS, EDS scans were conducted over the entire visible area of the field of view, and selected areas on the material surface were also scanned to confirm the distribution and proportion of elements on the surface.

### 4.5 Species Distribution Calculation

The professional thermodynamic calculation software PHREEQC (United States Geological Survey) was used to calculate the distributions of the chemical species of Pb(II) and Bi(III) in HBr solution. The calculations employed the Pitzer.dat database, which applies the Pitzer equations [33, 34] to calculate the activity coefficients of specific components in the solution. This method is suitable for high‐concentration brines. The stability constants of the Pb─Br and Bi─Br complexes were obtained from the literature [26, 27]: for Pb─Br complex, lgβ_1_ = 1.10, lgβ_2_ = 1.38, and lgβ_3_ = 2.38; for Bi─Br complex, lgβ_1_ = 2.36, lgβ_2_ = 4.41, lgβ_3_ = 6.26, lgβ_4_ = 7.70, lgβ_5_ = 8.30, and lgβ_6_ = 9.38. During the calculation, the concentrations of Pb(II) and Bi(III) were both set to 90 mg/L, consistent with the conditions of the batch experiments. Br^−^ was used to balance the charge of the system.

### 4.6 Density Function Theory Calculations

All quantum chemical calculations were performed using Gaussian 16 software within the DFT framework. Geometry optimizations were conducted in the gas phase employing the B3LYP [35] functional. Scalar relativistic effective core potentials (RECPs) [36, 37, 38] were used for Pb, Bi, and Th, with the ECP78MWB‐SEG [36] basis set replacing 78 core electrons for Pb and Bi, whereas the ECP60MWB‐SEG [37, 38] basis set was applied to Th, replacing 60 core electrons. The remaining light atoms were treated with the 6–31G(d) basis set. Frequency calculations at the same level of theory confirmed that all optimized structures represent local minima on the potential energy surface. Dispersion interactions were corrected using Grimme's DFT‐D3 [39] method. To enhance the reliability of energetic assessments and incorporate solvation effects, single‐point calculations were performed using a larger 6‑311G(d,p) basis set and the conductor‐like screening model [40, 41] at the B3LYP level. The adsorption of Br^−^ onto the cationic resin was simulated with n‐dodecane (ε = 2.006) as the solvent to mimic the low‐dielectric microenvironment, whereas water (ε = 78.355) was used for all other reactions. Solution‐phase Gibbs free energies were obtained by combining the gas‐phase thermally correction to the Gibbs free energy with the solvation energy obtained at the B3LYP/RECP/6‑311G(d,p) level [42]. ESP and IGMH analyses were conducted using Multiwfn 3.8 [43], and the results were visualized in Visual Molecular Dynamics (VMD) [44].

## Author Contributions

Y.W., S.N., X.Y., and L.S. conceived and directed the research. L.C. designed the experiments and wrote the manuscript. W.F., X.H., S.W. and Z.Z. carried out major experiments. W.L. and Q.W. contributed to the sample characterization. N.Z. contributed to species calculation of lead and bismuth. L.S. contributed to the DFT calculation. All authors discussed the results and gave approval to the final version of the manuscript.

## Funding

This work was supported by the National Natural Science Foundation of China [Grant No. 12405384, 22350710186, U23B20167], and the National Key R&D Program of China [2022YFB3506100].

## Conflicts of Interest

L.C., Y. W., S. N., X. Y., N. Z., X. H. and W. F. have filed a patent application covering the entire content of this study (patent applicant: University of South China; Inventors: Lifeng Chen, Yuezhou Wei, Shunyan Ning, Xiangbiao Yin, Ji Wang, Ningchao Zheng, Xuexiang He, Wannian Feng; application No.: PCT/ CN2025/097760). The other authors declare no competing interests.

## Supporting information




**Supporting File**: advs74721‐sup‐0001‐SuppMat.docx.

## Data Availability

The data that support the findings of this study are available in the supplementary material of this article.
