# Supplementary material for: Highly Efficient Extraction of 212 Pb/212Bi from the Decay Chain of 232Th‐Based on Anion Exchange in Bromide Medium
Source: Adv Sci (Weinh). 2026 Mar 9;13(28):e74721. doi: 10.1002/advs.74721 (PMC13185826; doi:10.1002/advs.74721)
Supplement: Supplementary file 1 — Supporting File: advs74721‐sup‐0001‐SuppMat.docx. [file ADVS-13-e74721-s001.docx]

**Highly Efficient Extraction of ^212^Pb/^212^Bi from the Decay Chain of ^232^Th Based on Anion Exchange in Bromide Medium**

*Lifeng Chen, Wannian Feng, Yuezhou Wei*, Xuexiang He, Shaoying Wang, Zhongyuan Zhou, Qiang Wu, Ningchao Zheng, Xiangbiao Yin, Wenlong Li, Lingling Su*, Shunyan Ning**

L. Chen, W. Feng, Y. Wei, X. He, S. Wang , Z. Zhou, Q. Wu, N. Zheng, X. Yin, W. Li, L. Su, S. Ning

*School of Nuclear Science and Technology, University of South China, 28 Changsheng West Road, Hengyang 421001, PR China.*
E-mail: [[*yzwei@usc.edu.cn*](mailto:yzwei@usc.edu.cn); *linglingsu@usc.edu.cn*](mailto:linglingsu@usc.edu.cn); [*ningshunyan@usc.edu.cn*](mailto:ningshunyan@usc.edu.cn)

L. Chen, W. Feng, Y. Wei, X. He

*Key Laboratory of Advanced Nuclear Energy Design and Safety, Ministry of Education, 28 Changsheng West Road, Hengyang 421001,* *PR China.*

Y. Wei

*Institute of Nuclear Fuel Cycle and Materials, School of Mechanical Engineering, Shanghai Jiao Tong University, Shanghai 200240, PR China*

*L. Su*

*Laboratory of Nuclear Energy Chemistry, Institute of High Energy Physics, Chinese Academy of Sciences, Beijing 100049, PR China*

Funding: This work was supported by the National Nature Science Foundation of China [Grant No. 12405384, 22350710186, U23B20167], and the National Key R&D Program of China [2022YFB3506100].

**S1 Parameter setting for batch experiments**

Here, some slant letters or symbols were used to denote the experimental parameters. The ratio of adsorbent mass (solid) to volume of solution (liquid) was represented as *S*/*L*; The contact time between adsorbent and solution was represented as *t*; The ambient temperature was denoted as *T*; The shaking frequency of water bath shaker was represented as *F*; The concentration of specific component M was represented as [M].

(1) Experiments for acidity

**Solution parameters**: *[Th] = 100 mg/L, [La] = 60 mg/L, [Pb] = 90 mg/L, [Bi] = 90 mg/L, [Ba] = 60 mg/L, medium: different concentrations of HCl, HBr or HI.*

**Experimental parameters:** *S/L = 0.05 g/20 mL,* *t = 2h, T = 25℃, F=120 rpm*

**Variables**: *[HCl] = 0.3, 0.5, 1.0, 1.5, 2.0, 3.0, 4.0 or 5.0 M; [HBr] = 0.3, 0.5, 1.0, 1.5, 2.0, 3.0, 4.0 or 5.0 M; [HI] = 0.5, 1.0, 1.5, 2.0, 3.0, 4.0 or 5.0 M.*

1. Experiments for adsorption kinetics

**Solution parameters**: *[Pb] or [Bi]= 90 mg/L, [HBr] = 0.5 M*

**Experimental parameters:** *S/L = 0.05 g/20 mL, T = 25℃, F =120 rpm*

**Variables:** *t = 3, 5, 10, 20, 30, 60, 90, 120, 180, 240 or 300 min*

(3) Experiments for adsorption isotherms

**Solution parameters**: *[HBr] = 0.5 M, different concentrations of Pb(II) or Bi(III)*

**Variables:**

*[Pb] = 50, 100, 150, 200, 250, 300, 350 or 400 mg/L.*

*[Bi] = 50, 100, 150, 200, 250, 300, 350, 400, 650, 750, 850 or 1000 mg/L.*

**Experimental parameters:** *S/L = 0.05 g/20 mL, T = 25℃, F=120 rpm, t = 1 h*

(4) Experiments for adsorption dynamics

**Solution parameters**: *[Pb] or [Bi]= 90 mg/L, [HBr] = 0.5 M*

**Experimental parameters:** *S/L = 0.05 g/20 mL, F =120 rpm, t = 1 h*

**Variables:** *T = 25, 35, 45, 55 or 65℃*

**S2 Parameter setting for column experiments**

Here, some slant letters or symbols were used to denote the experimental parameters. The column dimensions including inner diameter and height were denoted as *φ* × *h*; The flow speed for adsorption, rinse and elution were denoted as *f_a_*, *f_r_* and *f_e_*, respectively. The dead volume of the whole column system was denoted as *D.V.*; The volume of solution was denoted as *V*.

(1) Breakthrough experiments for Pb(II) and Bi(III)

**Experimental parameters:** *column φ × h = 10 mm × 100 mm, f_a_ = 10 or 20 mL**/min*

**Solution parameters and feed order:**

① *Working solution: [Pb] or [Bi] = 200 mg/L, [HBr] = 0.5 M, V = 5000 mL*

(2) Dynamic desorption experiments for Pb(II)

**Experimental parameters:** *column φ × h = 10 mm × 100 mm, D.V. = 16.6 mL, f_a_ = 5.0 mL/min, f_e_ = 2.0 mL/min*

**Solution parameters and feed order:**

① *Working solution: [Pb] = 25 mg/L, [HBr] = 0.5 M, V = 100 mL*

② *Eluant: UPW, 0.01 M HCl or 1.0 M HNO_3_, V = 200 mL*

(3) Dynamic desorption experiments for Bi(III)

**Experimental parameters:** *column* φ *× h = 10 mm × 100 mm, D.V. = 16.6 mL, f_a_ = 5.0 mL/min, f_e_ = 2.0 mL/min*

**Solution parameters and feed order:**

① *Working solution: [Bi] = 25 mg/L, [HBr] = 0.5 M, V = 100 mL*

② *Eluant: 1 M HNO_3_ or 3.0 M HNO_3_, V = 200 mL*

(4) Separation of Pb and Bi from the simulated decay chain of thorium

**Experimental parameters:** *column* φ *× h = 10 mm × 100 mm, D.V. = 16.6 mL, f_a_ = 5.0 mL/min, f_r_ = 2.0 mL/min, f_e_ = 2.0 mL/min*

**Solution parameters and feed order:**

① *Working solution: [Pb] = 20 mg/L, [Bi] = 20 mg/L, [Th] = 20 mg/L, [Ba] = 20 mg/L, [La] = 20 mg/L, [HBr] = 0.5 M, V = 500 mL*

② *Rinse reagent: 0.5 M HBr, V = 50 mL*

③ *Eluant 1: UPW, V = 200mL*

④ *Eluant 2: 0.1 M HCl, V = 100 mL*

⑤ *Eluant 3: 1.0 M HNO_3_, V = 100 mL*

(5) Amplified experiment for Pb separation from simulated decay chain of thorium-232

**Experimental parameters:** *column* φ *× h = 35 mm × 300 mm, D.V. = 196 mL, f_a_ = 50 mL/min, f_r_ = 10 mL/min, f_e_ = 10 mL/min*

**Solution parameters and feed order:**

① *Working solution: [Pb] = 5 mg/L, [Bi] = 5 mg/L, [Th] = 180 mg/L, [Ba] = 5 mg/L, [La] = 5 mg/L, [HBr] = 0.5 M, V = 5000 mL*

② *Rinse reagent: 0.5 M HBr, V = 500 mL*

③ *Eluant: 1.0 M HNO_3_, V = 1000 mL*

(6) Real ^212^Pb separation from the decay chain of thorium-232

**Experimental parameters:** *column* φ *× h = 35 mm × 300 mm, D.V. = 196 mL, f_a_ = 50 mL/min, f_r_ = 10 mL/min, f_e_ = 10 mL/min*

**Solution parameters and feed order:**

① *Working solution: 2 kg Th(NO_3_)_4_·xH_2_O (old thorium) dissolved in 5 L 0.5 M HBr, Activity of ^212^Pb = 2.37 MBq.*

② *Rinse reagent: 0.5 M HBr, V = 500 mL*

③ *Eluant: 1.0 M HNO_3_, V = 1000 mL*

(7) Dynamic adsorption-desorption cycle experiments

**Experimental parameters:** *column* φ *× h = 5 mm × 50 mm, f_a_ = 1.0 mL/min, f_e_ = 10 mL/min*

**Solution parameters and feed order:**

① *Rinse solution for column pretreatment*: *[HBr] = 0.5 M, V = 50 mL*

② *Working solution: [Pb] = 427 mg/L, [Th] = 1000 mg/L, [HBr] = 0.5 M*

③ *Eluant: 1.0 M HNO_3_, V = 50 mL*

*The feed procedure was repeated for 4 times.*

**S3 Cost estimation for SiPyR-N4**

The cost estimation for 1 kg SiPyR-N4 is detailed as follows:

SiO₂: 500 CNY/kg × 1 kg = 500 CNY

4-Vinylpyridine: 1600 CNY/250 mL × 200 mL = 1280 CNY

Divinylbenzene: 144 CNY/500 mL × 40 mL = 11.52 CNY

Other auxiliary reagents (e.g., diethyl phthalate, acetone, acetophenone): ≈ 100 CNY

Labor and utilities: ≈ 100 CNY

Total cost: 500 + 1280 + 11.52 + 100 + 100 = 1991.52 CNY

**S4 Some experimental results**

**Table S1** Parameters obtained by fitting the kinetic data with PFO and PSO model

| Metal kinds | *Q_e_* _(_ (mg/g) | PFO | | | PSO | | |
| --- | --- | --- | --- | --- | --- | --- | --- |
|  |  | *Q_est_*  (mg/g) | *k*_1_  (min^−1^) | *R*^2^ | *Q_est_*  (mg/g) | *k*_2_  (mg·g^−1^·min^−1^) | *R*^2^ |
| Pb^2+^ | 32.1 | 31.5 | 0.507 | 0.990 | 32.1 | 0. 211 | 1.00 |
| Bi^3+^ | 36.0 | 35.4 | 0.432 | 0.988 | 36.1 | 0.195 | 1.00 |

**Table S2** Parameters obtained by fitting the data of adsorption isotherms of Pb(II) with the Langmuir model and the Freundlich model

| Langmuir model | | | |  | Freundlich model | | |
| --- | --- | --- | --- | --- | --- | --- | --- |
| *Q_max_* (mg/g) | *K_L_*(L/mg) | | R^2^ |  | *K_F_* | *N* | *R*^2^ |
| 206 | 0.0171 | 0.990 | |  | 21.6 | 2.81 | 0.916 |

**Table S3** Parameters obtained by fitting the data of adsorption isotherms of Bi(III) with the Langmuir model and the Freundlich model

| Langmuir model | | |  | Freundlich model | | |
| --- | --- | --- | --- | --- | --- | --- |
| *Q_m_* (mg/g) | *K_L_*(L/mg) | *R*^2^ |  | *K_F_* | *n* | *R*^2^ |
| 125 | 0.394 | 0.990 |  | 40.5 | 4.60 | 0.952 |

**Table S4** Parameter obtained by fitting the thermodynamic data with the Van’t Hoff equations

| Metal cation | Δ*H* (kJ/mol) | Δ*S* (J/(K·mol)) | Δ*G* (kJ/mol) (25℃) |
| --- | --- | --- | --- |
| Pb^2+^ | -37.6 | -59.4 | -20.0 |
| Bi^3+^ | -12.7 | 47.0 | -26.8 |

**Table S5** Atomic percent of different elements on the surface of SiPyR-N4 before and after the adsorption of Pb or Bi

| Sample name | Location | C(%) | N(%) | O(%) | Si(%) | Br(%) | Pb(%) | Bi(%) | Total(%) |
| --- | --- | --- | --- | --- | --- | --- | --- | --- | --- |
| SiPyR-N4-Br | A | 26.96 | 2.92 | 44.35 | 22.97 | 2.80 | 0 | 0 | 100 |
| SiPyR-N4-Pb | B | 24.27 | 1.98 | 43.17 | 24.30 | 4.72 | 1.56 | 0 | 100 |
| SiPyR-N4-Bi | C | 23.60 | 2.29 | 49.70 | 19.64 | 4.00 | 0 | 0.76 | 100 |

**Table S6** Reaction energetics of the cation resin MBP⁺ with bromo-species.

| Reactions | ∆G (kcal/mol) |
| --- | --- |
| MBP^+^ + Br^-^ → MBPBr | -32.0 |
| MBPBr + PbBr_3_^-^ → MBP(PbBr_3_) + 3Br^-^ | -10.8 |
| 2MBPBr + BiBr_5_^2-^ → (MBP)_2_(BiBr_5_) + 2Br^-^ | -17.8 |
| 3MBPBr + BiBr_6_^3-^ → (MBP)_3_(BiBr_6_) + 3Br^-^ | -29.4 |

**Table S7** Gibbs free energies for the hydration and bromo-complex formation of metal ions.

| Reactions | ∆G (kcal/mol) |
| --- | --- |
| Pb^2+^+ 3Br^-^ = PbBr_3_^-^ | -72.9 |
| Bi^3+^+ 5Br^-^ = BiBr_5_^2-^ | -239 |
| Bi^3+^+ 6Br^-^ = BiBr_6_^3-^ | -240 |
| [Th(H_2_O)_8_]^4+^ + H_2_O = [Th(H_2_O)_9_]^4+^ | -9.79 |
| [Th(H_2_O)_8_]^4+^ + Br^-^ = [Th(H_2_O)_7_Br]^3+^+ H_2_O | -6.14 |

All values correspond to the Gibbs free energy change for the reaction as written, with all species in aqueous solution.

**Table S8** Comparison of different methods for the acquisition of ^212^Pb

| Acquisition method | principle | Core materials | Chemical yield (%) | Radionuclide purity | Cost | Safety | Potential scalability | Ref. |
| --- | --- | --- | --- | --- | --- | --- | --- | --- |
| ^228^Th/^224^Ra/^212^Pb generator | Gas diffusion | \ | 70 | H | H | H | L | [1] |
| ^224^Ra/^212^Pb generator | Cation exchange | cation exchange resin | ≥95 | H | H | H | L | [2] |
| ^212^Pb extraction from thorium by Pb-resin^TM^ | Solid-phase extraction + Supramolecular recognition | DtBuDC18C6 | 83.0 | H | M | M | M | [3] |
| ^212^Pb extraction from thorium by DSADB18C6 | Liquid-liquid extraction + Supramolecular recognition | DSADB18C6 | 85.7 | \ | M | M | M | [4] |
| ^212^Pb extraction from thorium by DtBuDC18C6 | Cloud point extraction + Supramolecular recognition | DtBuDC18C6 | 53.8 | \ | M | M | M | [5] |
| ^212^Pb extraction from thorium by SiPyR-N4 | Anion exchange + species regulation (HCl) | SiPyR-N4 | \ | \ | L | M | M | [6] |
| ^212^Pb extraction from thorium by SiPyR-N4 | Anion exchange + species regulation (HBr) | SiPyR-N4 | 88.3 | H | L | M | H | This work |

Note: “\” represents none or not mentioned; H, M, L denote high, middle and low, respectively; The potential scalability was evaluated by the cost. Higher cost means lower potential scalability.


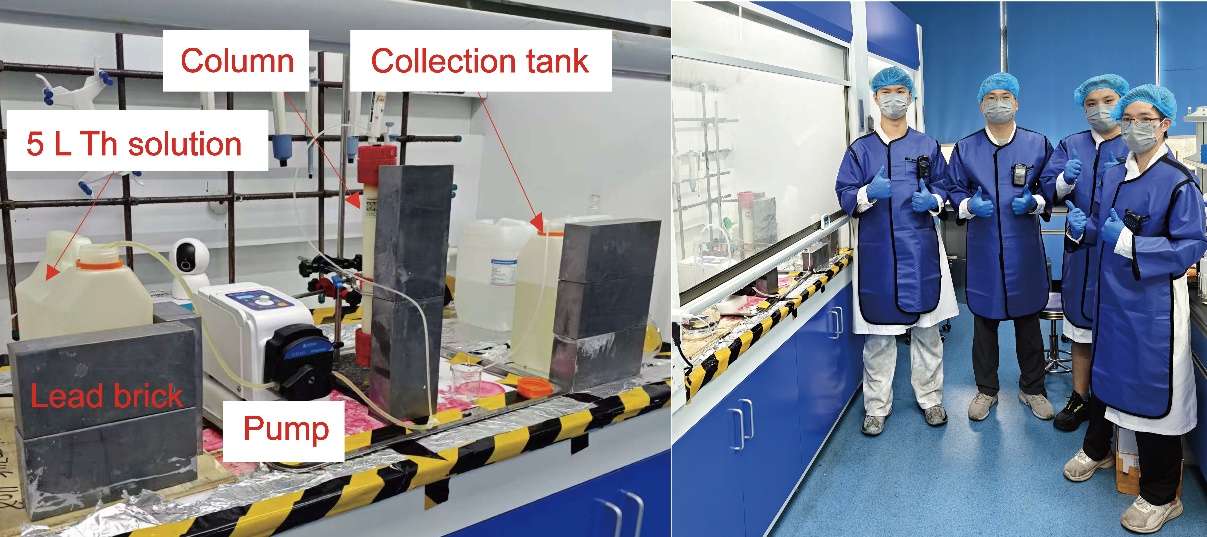


**Figure S1 On-site experimental devices (left) and experimenters (right) for the hot separation**


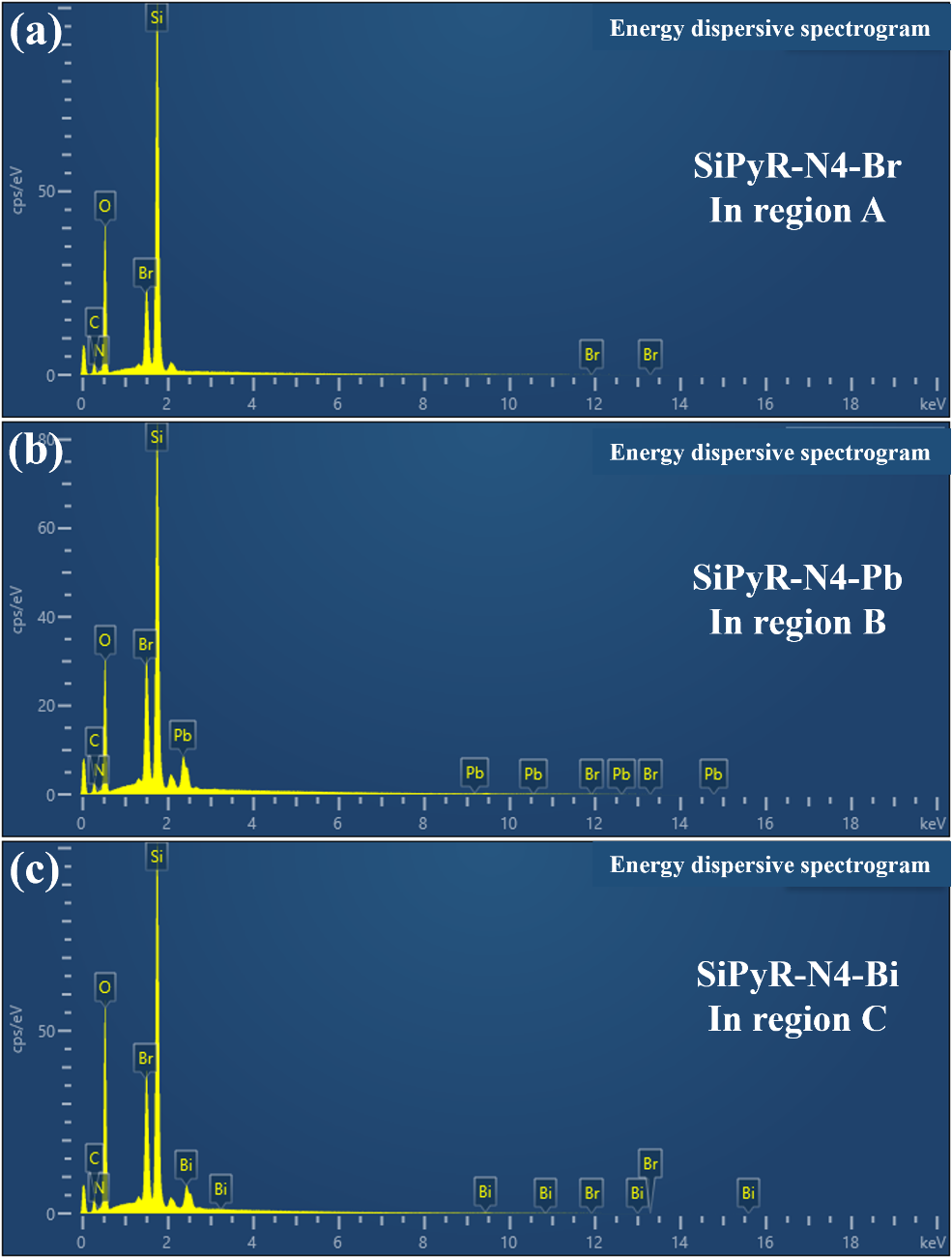


**Figure S2 Total distribution map of spectrograms of different samples**. **a** SiPyR-N4-Br. **b** SiPyR-N4-Pb. **c** SiPyR-N4-Bi


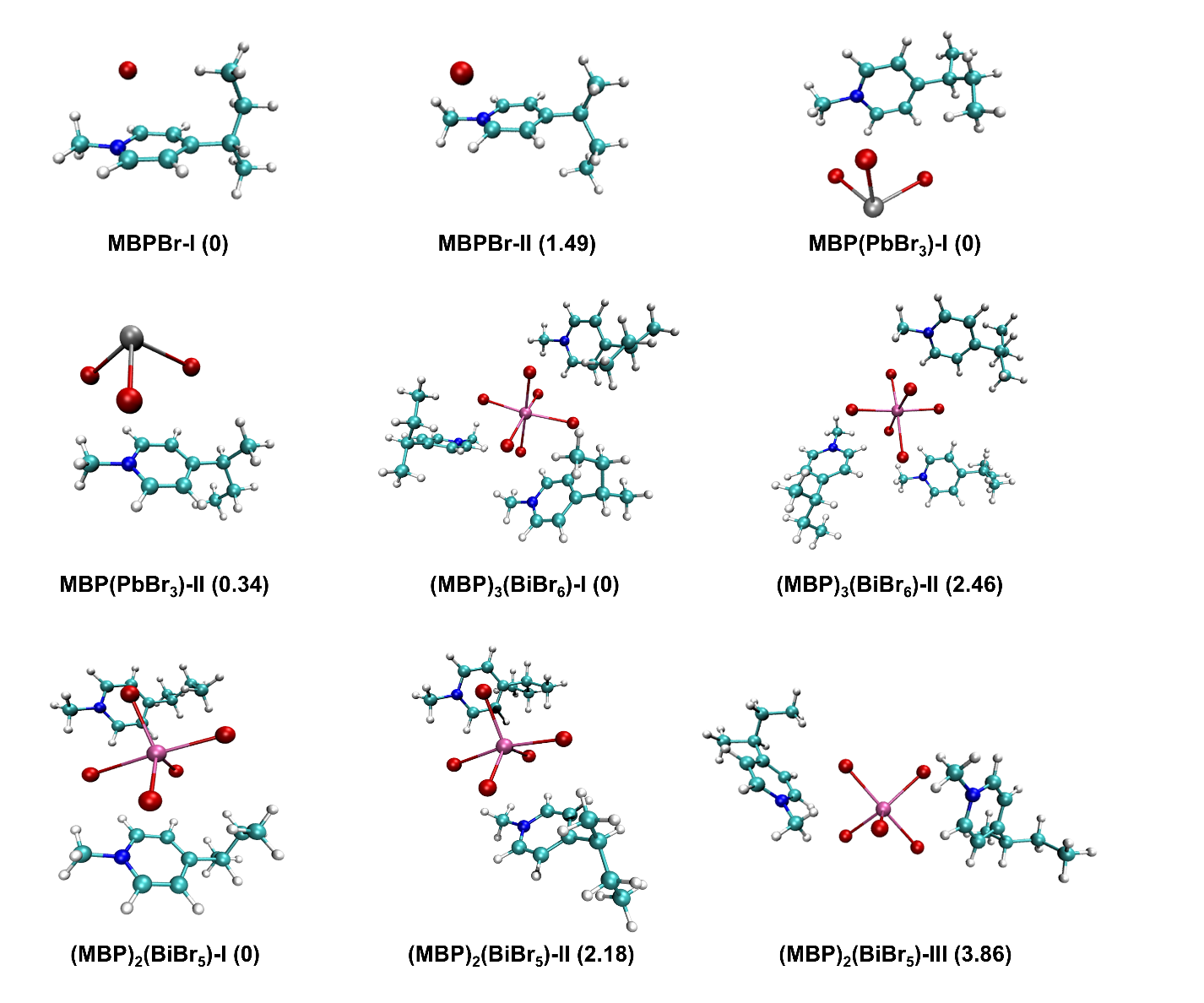


**Figure S3 Structural configurations and relative energies of MBP^+^ with different kinds of anion.** Optimized molecular structures and corresponding relative Gibbs free energies (in kcal/mol) for different complexes formed between the MBP+ cationic resin and the indicated anions. The most stable configuration is set as the energy zero.


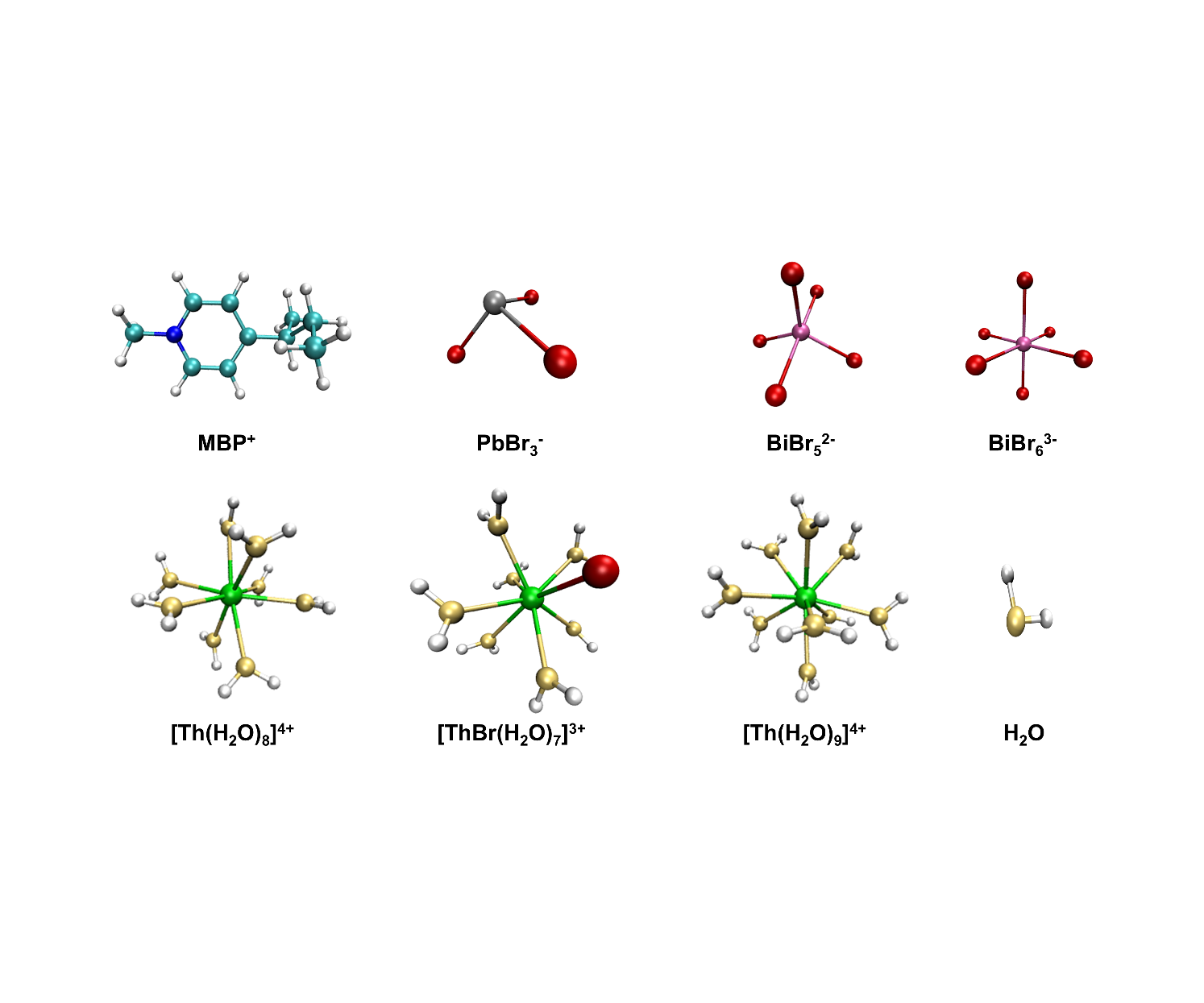


**Figure S4 Additional chemical species investigated in this study.** Optimized geometries of other relevant structures analyzed in this work.

**References**

[1] S. Hassfjell, A ^212^Pb generator based on a ^228^Th source, Applied Radiation and Isotopes 55 (2001) 433-439.

[2] M. Pruszynski, R. Walczak, M. Rodak, et al., Radiochemical separation of ^224^Ra from ^232^U and ^228^Th sources for ^224^Ra/^212^Pb/^212^Bi generator, Applied Radiation and Isotopes 172 (2021) 109655.

[3] J. Chen, M. Xu, Y. Liu, et al., Isolation of ^212^Pb from natural thorium for targeted alpha-therapy, Chinese Chemical Letters 33 (2022) 3474-3477.

[4] S. Cao, Y. Kang, H. Tang, et al., Separation of lead-212 from natural thorium solution utilizing novel sulfonamide dibenzo-18-crown-6, Dalton Transactions 53 (2024) 3722-3730.

[5] S. Cao, B. Hao, Z. Chen, Crown ether-based cloud point extraction for lead-212 enrichment from thorium series, New Journal of Chemistry 49 (2025) 1342-1348.

[6] X. He, W. Feng, Z. Wang, et al., An advanced separation method for the acquisition of 212Pb/212Bi from natural thorium, Chemical Engineering Journal 502 (2024) 157971.
